# Neurodevelopmental Differences, Pedohebephilia, and Sexual Offending: Findings from Two Online Surveys

**DOI:** 10.1007/s10508-021-02228-w

**Published:** 2022-01-06

**Authors:** Sara Jahnke, Alexander F. Schmidt, Andrea Klöckner, Jürgen Hoyer

**Affiliations:** 1 Department of Health Promotion and Development, University of Bergen, 5020 Bergen, Norway; 2 Department of Psychology, Social & Legal Psychology, Johannes Gutenberg-Universität Mainz, Mainz, Germany; 3 Institute for Clinical Psychology and Psychotherapy, Technische Universität Dresden, Dresden, Germany

**Keywords:** Pedophilia, Child sexual abuse, Neurobiology, Neurodevelopment, Etiology, DSM-5

## Abstract

The neurodevelopmental theory of pedohebephilia states that sexual interests in children arise from early neurodevelopmental perturbations, as, for example, evidenced by increased non-right-handedness, more childhood head injuries, and reduced intelligence and height. As corroborating evidence largely rests on samples of convicted men, we conducted online surveys among German-speaking (Study 1, *N* = 199) and English-speaking men (Study 2, *N* = 632), specifically targeting community members with pedohebephilic or teleiophilic interests. Although we detected theoretically meaningful sexual interest patterns in an embedded viewing time task, we could not detect expected neurodevelopmental differences between teleiophilic and pedohebephilic men in either of the two studies. Strikingly, pedohebephilic men who reported convictions for sexual offenses emerged as shorter and less intelligent than pedohebephilic men without convictions in Study 2. While elucidating possible third variable confounds, results have to be interpreted cautiously because of the methodological problems inherent to non-matched case control designs.

**Supplementary Information:**

The online version contains supplementary material available at 10.1007/s10508-021-02228-w.

## Introduction

In order to solve the puzzle of what may cause pedophilia or hebephilia, that is, adult men's sexual attraction to sexually immature pre- or pubescent partners, respectively (Seto, 2012), numerous studies have compared neurodevelopmental differences between participants with and without pedophilia (see Tenbergen et al., 2015 for an overview). For example, neuroimaging techniques revealed structural or functional differences in the brains of pedophilic and teleiophilic (i.e., sexually attracted to adults) participants (e.g., Cantor et al., 2008; Poeppl et al., 2011, 2015). Furthermore, several studies found an association between pedophilia and soft markers for early neurodevelopmental perturbations, such as non-right-handedness, height, IQ, or head injuries before age 13 (Blanchard et al., 2002, 2007; Cantor et al., 2007, 2005a,b; Fazio, 2018). Yet, as the majority of studies are mostly restricted to samples of men who have sexually offended, it is problematic that some of these factors are also known markers of criminal behavior (e.g., lower height and intelligence, Beckley et al., 2014; non-right-handedness, Bogaert, 2001; early head injuries, Liu, 2011). Adding the fact that there are many men with pedohebephilic interests who refrain from offending and are socially well adapted (Joyal et al., 2019), it is questionable whether these differences really are due to patterns of sexual attraction and not to other factors that are associated with (having been or) being arrested or convicted. To rule out such an alternative interpretation, a replication of neurodevelopmental differences between participants with and without pedophilia in community samples seems mandatory (Joyal et al., 2007).

For solving the cardinal problem of recruiting non-institutionalized participants with an uncommon and stigmatized sexual attraction pattern such as pedophilia or hebephilia, the anonymity of online studies might be particularly helpful. Following this logic, we sought to compare markers for neurodevelopmental differences among men with different sexual maturity interests in German-speaking (Study 1) and English-speaking (Study 2) online samples. With a combined sample of 831 participants, we hope to extend the currently limited evidence base concerning the relationship between neurodevelopmental differences, pedohebephilia, and offending behavior.

### A Critical Discussion of the Early Neurodevelopmental Perturbations Hypothesis

Currently, pedohebephilic interest is increasingly viewed as being caused by an “underlying brain dysfunction, one that prevented the development of more typical intellectual and sexual characteristics” (Cantor et al., 2005a, 2005b, p. 448). Neurodevelopmental deficits can be caused by genetic disorders, accidents, brain tumors, and pre- or postnatal exposure to teratogenic/toxic substances in utero (Becerra García, 2009). While a variety of such perturbations can have severe and long-lasting effects on cognitive functioning, they also manifest as "very mild, often inconsequential features that are associated with atypical neurodevelopment" (Fazio, 2018, p. 1205), such as non-right-handedness, undetached ear lobes, or other biomarkers (see Jordan et al., 2020 for a critical overview). Empirical research supporting the neurodevelopmental perturbations hypothesis of pedohebephilia has mostly been conducted on men who have been referred to clinical institutions "as a result of illegal or clinically significant sexual behaviors or interests" (Cantor et al., 2007, p. 397). Based on those samples, findings from several neuroimaging studies indicated structural abnormalities in the brains of pedophilic men who have sexually offended compared to forensic or non-forensic teleiophilic controls (Cantor et al., 2008; Schiffer et al., 2007). Researchers have also reported higher rates of non-right-handedness (Blanchard et al., 2007; Cantor et al., 2005a, 2005b), lower height (Cantor et al., 2007; Fazio et al., 2017), more head injuries during childhood (Blanchard et al., 2002, 2003), more minor physical abnormalities (Dyshniku et al., 2015), and lower IQs (Blanchard et al., 2007) among forensic/clinical samples of pedohebephilic men. Notably, data on soft markers for neurodevelopmental perturbations especially need replication, because they were mostly obtained in the same institution (Centre for Addiction and Mental Health, Toronto) from members of the Kurt Freund Laboratory. All studies were conducted on (potentially overlapping samples of) cases referred mostly for forensic evaluations (see, e.g., Blanchard et al., 2002), precluding the recruitment of larger numbers of pedohebephilic men who have not offended.

Hebephilia is not a universally accepted concept, and the proposition to include sexual interests in pubescents as a disorder category was not adopted in the DSM-5 (Singy, 2015). Yet, irrespective of whether hebephilic interests can be considered pathological, the concept has demonstrated its merit for research purposes. There are good reasons to assume that sexual attraction to prepubescents and to pubescents have similar causes, since the course of sexual maturation leads to gradual changes in physical appearance from a pre-pubescent to a pubescent and finally a postpubescent state (Blanchard et al., 2007; see also Stephens et al., 2017 for a discussion of similar arousal patterns among hebephilic and pedophilic men who have sexually offended). Empirically, hebephilic men have sometimes been found to show IQ scores, rates of right-handedness, and childhood brain injuries that are intermediate between those obtained among pedophilic and teleiophilic men in forensic samples (Blanchard et al., 2003, 2007; Cantor et al., 2005a, 2005b).

### Potential Biases Associated with Studying Pedohebephilia in Clinical or Forensic Samples

Prior studies attempted to control for factors associated with criminality by comparing people with pedohebephilia with teleiophilic people, who have also mostly been referred because of criminal or disturbing sexual activities (e.g., Blanchard et al., 2002; Fazio et al., 2017). Yet, just as increased levels of antisociality among teleiophilic men who have sexually offended would not lead us to suppose that being antisocial is a characteristic feature of teleiophilia, the problem remains that the detected markers for neurodevelopmental perturbations may be more relevant for pedohebephilic men who sexually offended than for pedohebephilia per se. Some researchers have therefore cautioned that "it seems far-fetched to associate pedophilia with neurodevelopmental disorders" (Joyal et al., 2019, p. 154) as long as unequivocal empirical support for such an account is lacking.

Additionally, some neurodevelopmental markers discussed as being specific for pedophilia, such as increased rates of left handedness/ambidexterity, have also been found among men with non-heterosexual orientation (Lalumière et al., 2000) or asexuality (Yule et al., 2014). Moreover, non-right-handedness has been linked to alcohol consumption (Denny, 2011) and a plethora of other psychological disorders such as posttraumatic stress disorder (Boscarino & Hoffman, 2007), depression (Denny, 2009), or anorexia nervosa (Tenconi et al., 2010), to name a few. Lower IQ, lower fraternal birth order, neuropsychiatric abnormalities, non-right-handedness, and lower D2/D4 length are also associated with sexual offending in general, non-sexual delinquency, thrill seeking proclivity, and aggressive traits (Bailey & Hurd, 2005; Bogaert, 2001; Brower & Price, 2001; Fink et al., 2006; Moffitt et al., 1994; Ogunfowokan et al., 2016). These findings underscore the unspecificity of the neurodevelopmental markers that have been associated with pedohebephilia, and invite speculation whether the differences between pedohebephilic men who have sexually offended and teleiophilic control groups may be attributable to variables other than sexual interest. The same problems apply to studies that compare pedohebephilic men from treatment settings with healthy teleiophilic men, as the former group is likely to experience more distress about their sexuality or more concerns about sexual offending than pedohebephilic men who did not seek help. In a recent Swedish study, Abé et al. (2021) compared 55 help-seeking men with pedophilic disorder from a national helpline with 57 age-matched controls recruited via the institute homepage and university services. The authors also report significant differences between the brains of pedophilic patients and controls, which, for the most part, remained significant when excluding patients who had committed either sexual abuse or child pornography offenses, indicating that these findings are not explained solely by criminality or antisociality. Furthermore, pedophilic disorder was linked to a lower IQ, higher rates of several mood and anxiety disorders, and antisocial personality disorders, while the study found no differences regarding handedness and height (with pedophilic men emerging descriptively as taller and less likely to be non-right-handed).

### Neurodevelopmental Perturbation Markers in Community Samples of Pedohebephilic Men

About 50% or more of sexual offenses against children are committed by teleiophilic men (Schmidt et al., 2013; Seto, 2008), whereas many pedophilic men never commit sexual offenses at all (Dombert et al., 2016; Joyal & Carpentier, 2021). Pedohebephilic men in community settings tend to show better psychological adjustment than those recruited in clinical or forensic environments (Jahnke et al., 2015). Recently, an important first step toward disentangling factors associated with sexual offending and pedohebephilic attraction has been made. Based on data from the German research project Neural Mechanisms underlying Pedophilia and Child Sexual Abuse (NeMUP), Gerwinn et al. (2018) found that "many of the factors reported as being related to paedophilia may […] actually be associated with committing child sex offences and vice versa" (p. 75). In their sample, pedophilic and non-pedophilic men did not differ with regard to handedness, accidents leading to unconsciousness in childhood, or IQ. Yet, with 155 pedophilic participants (about half of which had never committed sexual offenses), a larger evidence base is needed to achieve sufficient test power to corroborate the purported small effect sizes of links between pedohebephilia and markers for neurodevelopmental impairment, as the authors point out themselves. Regarding further indicators of neurodevelopmental differences, results based on participants in the NeMUP project revealed similar results, namely that structural abnormalities in the brain (Schiffer et al., 2017) and impairments in executive functioning (Massau et al., 2017) are linked to offending status but not sexual interests (cf. Abé et al., 2021 who found that most brain differences remained significant when controlling for the sexual offending status of their pedophilic participants).

### The Present Studies

In the following studies, we sought to test selected indicators of neurodevelopmental differences within two community samples, focusing solely on markers that are accessible in an anonymous online setting. Prior studies on the neurodevelopmental correlates of pedophilia among forensic samples typically classified participants as pedohebephilic either based on offense behavioral indicators as proxies (e.g., Cantor et al., 2005a, 2005b) or on participants’ responses in a phallometric test or their self-reported sexual interests (e.g., Blanchard et al., 2002, 2003; Fazio et al., 2017). In the present studies, we employed self-report and viewing time (VT) measures to assess sexual maturity interests. The VT measure was chosen as an objective measure (Schmidt et al., 2017) for cross-validation of the self-report.

By not relying on expert diagnosis of pedophilic disorder, we avoided classifying participants based on categorical disorder diagnoses which in the case of pedophilia suffer from particularly high uncertainty (i.e., roughly between one- and two-thirds of pedophilia diagnoses may be considered wrong due to low interrater reliability, Mokros et al., 2018). Our community data are also less likely to be confounded by third variables that are associated with caseness status. This is a typical problem of case–control designs, whereby individuals who by definition need to show clinically significant impairment in terms of subjective distress or interpersonal functioning are compared to persons who are unlikely to exhibit signs of distress or functioning deficits. Yet, note that non-matched case–control studies such as the present ones are tainted by other potential biases, which we will discuss in limitations section.

#### Hypotheses

Above and beyond testing whether VT paradigms were valid in a specific online environment, we will test the following hypotheses based on the discussed limitations of the empirical evidence for the neurodevelopmental perturbation hypothesis:

1. In case the neurodevelopmental perturbation hypothesis will be specific for pedohebephilic sexual interests, pedohebephilic men will differ from teleiophilic men (i.e., men with a sexual interest in sexually mature individuals) with respect to markers of neurodevelopmental perturbations in community samples.
2. In case of a confound with self-reported sexual offending status, pedohebephilic men who have sexually offended will differ from pedohebephilic men who have not offended with respect to markers of neurodevelopmental perturbations.

We will test the following markers for neurodevelopmental perturbations: (a) height, (b) IQ, (c) non-right-handedness, and (d) injuries resulting in unconsciousness before and after age 13, all of which will be tested as separate hypotheses. Study 1 was conducted among German-speaking individuals. In Study 2, we improved the assessments of IQ and handedness while repeating the study in a considerably larger English-speaking sample.

## Study 1

### Method

#### Participants and Procedure

All participants were recruited on websites, blogs, and web forums to participate in a study on neurological developmental problems and traumatic childhood experiences. No compensation was offered. Pedohebephilic individuals were sampled from websites for people with such sexual interests (jungsforum.net, schicksal-und-herausforderung.de, krumme13.org, boylandonline.com, Deutsches Girlloverforum, ITP-Arcados), while teleiophilic participants were recruited on science/psychology-related websites (e.g., forschung-erleben.uni-mannheim.de, caz-lesen.de, psychologie-heute.de, Facebook group "Psychologische Studien für alle"). We attempted to collect as much information as possible in a German-speaking sample. We stopped data collection when participation rates became very low. We did not attempt to reach a specific number of participants, as it was clear to us prior to the study that a statistically sufficient number of participants in German forums only will be unrealistic (see Table 1 for sample size calculations). In contrast to the larger-scale Study 2, Study 1 was conducted to test the feasibility of the online design. Thus, we focused more on the descriptive point estimates of effect sizes than significance levels. After we removed responses from 16 self-reported female participants and two participants who did not report their sex, the dataset contained 199 participants. Detailed information on how we categorized participants based on their sexual interests and offending status is presented in a separate section after the description of our measures. The study was approved by the ethical review board of the Technische Universität Dresden.

**Table 1:** Sample size calculations

Marker for neurodevelopmental deficits	Source	Effect size	Comparison group	Required sample size (two-tailed testing)
Height	McPhail and Cantor (2015)	*d* = .20 *d* = .21	Teleiophilic vs. pedohebephilic men Teleiophilic vs. pedophilic men	788 (one-tailed: 620) 714 (one-tailed: 564)
Handedness	Cantor et al. (2004)	*d* = .25 ^a^ *d* = .50 ^a^	Teleiophilic vs. pedohebephilic men Teleiophilic vs. pedophilic men	506 128
Head injuries before age 13	Blanchard et al. (2003)	*r*_s_ = .12	None, pedophilic, hebephilic, and teleiophilic participants were treated as an ordered set	542
IQ (Wechsler Adult Intelligence Scale)	Cantor et al. (2004)	*d* = .32 ^a^ *d* = .53 ^a^	Teleiophilic vs. pedohebephilic men Teleiophilic vs. pedophilic men	310 114

Calculations conducted with G*Power (for all calculations: 1–β = .80, α = .05, for height, handedness and IQ: *t* test, means: difference between two independent means, two groups: allocation ratio, N2/N1 = 1, for head injuries: exact, correlation, bivariate normal model: ρ H0 = 0, note that Spearman and Pearson correlations are computationally identical)

^a^ Calculated from summary statistics in Table 1 and in-text (Cantor et al., 2004, p. 7), *d* = *M*_1_–*M*_2_ / *SD*_pooled_

#### Measures

Participants filled out the scales in the order presented below. We included additional questionnaires to assess interests in sadomasochism, traumatic childhood experiences, and non-negative sexual experiences in childhood that will be featured in a separate publication (Jahnke et al., 2021). We were limited in our choice of intelligence measures, as most validated IQ scales are restricted to offline use by copyright holders. Therefore, we used a scale to assess crystallized intelligence (Schipolowski et al., 2014) as the only validated German scale that was, to our knowledge, available for online use at that time. We also developed a digit span test based on corresponding working memory tests in the Wechsler Adult Intelligence Scale (Lichtenberger & Kaufman, 2009). Yet, because of severe validity concerns regarding these two measures, we will not report their results.^1^ We calculated Cronbach’s α based on all of the 199 male participants in the dataset.

##### Viewing Time Measure of Sexual Interest

We presented five male and five female pictures of prepubescent children (Tanner stage 1; Tanner, 1990) and postpubescent adults each (Tanner stage 5) from the Not Real People Set (i.e., computer-generated pictures of Caucasian individuals in bathing suits provided by the Pacific Psychological Assessment Cooperation, 2004) in randomized order. Participants were prompted to indicate their attraction in a forced choice format with two response alternatives within a one second response window: "Yes, this is a potential sexual partner for me" vs. "No, this is not a potential sexual partner for me" (i.e., speeded VT task, Imhoff et al., 2010, Study 3, but note that we did not exclude trials with longer reaction times). We chose to instruct speeded responding to prevent participants from producing too many outliers due to unrestricted response windows in an online survey. Response latencies were recorded unobtrusively. For further details on how we dealt with statistical outliers see the section on VT classifications. Viewing time measures of pedohebephilia have repeatedly been shown to produce reliable (Welsch et al., 2021) and valid results (Pedneault et al., 2021; Schmidt et al., 2017).

##### Self-Reported Sexual Interests

A six-item scale introduced by Jahnke and Malón (2019) was administered to assess pedophilic, hebephilic, and teleiophilic interests by collecting attraction ratings on a Likert scale ranging from 1 (no sexual interest) to 10 (maximum sexual interest) with regard to children of each sex before puberty, adolescents of each sex in early stages of puberty, and sexually mature adult men and women.

##### Adapted Edinburgh Handedness Inventory

We used the modified version of the Edinburgh Handedness Inventory (EHI, Dyshniku et al., 2015). Participants had to decide whether they preferred the left or right hand for presented activities such as writing or drawing (forced choice). Due to a technical mistake, it was not possible to choose both the left and the right hand in cases where participants did not have a clear preference (ambidexterity, note that this mistake was fixed in Study 2). Like Dyshniku et al. (2015), we removed the items “using a broom” and “opening a box” because of poor factor analytic loadings. Based on the responses to the eight remaining items (α = 0.96), we computed a laterality quotient by dividing the difference score of left and right responses through the sum of left and right responses. Higher scores indicate a stronger preference for the right hand.

##### Head Injuries

Participants indicated how many head injuries leading to unconsciousness they had sustained before and after age 13. To prompt participant's memories, we translated and adapted questions from Blanchard et al. (2003), replacing "hockey" with "soccer" as a more popular sports activity among German children: "Were you ever knocked unconscious during your childhood (before you were 13 years old), for example, falling from a tree or hitting your head in a soccer game?" and "Were you ever knocked unconscious in adulthood (after 13 years of age), for example, in a car accident or in a sports injury?".

##### Social Desirability

Tendencies to give socially desirable responses were assessed with an eight-item German scale by Ray (1984). Participants were asked to respond to questions like “Are you quick to admit making a mistake?” with either "true" or "false." Higher values on the scale average indicate a higher risk for socially desirable responding (α = 0.67).

##### Sociodemographic Information (Including Height and Previous Convictions)

We assessed participant sex (male, female, other), age, educational achievement, and height on a cm scale. One participant (with stronger self-reported sexual attraction to adults than to children or adolescents) reported a height of 78 cm, which we corrected to the more likely value of 178 cm. Previous convictions for child sexual abuse, rape, or possession of child sexual exploitation material were assessed on a binary scale (yes/no) with three separate items (“I have been convicted for child sexual abuse,” “I have been convicted for rape,” “I have been convicted for child pornography offenses”).

### Categorizing Participants’ Sexual Interests and Offending Status

#### Classification Based on Self-Reported Sexual Attraction

In the pedophilic subsample, we retained only men who reported more sexual attraction to prepubescent children than to early-to-mid-pubertal adolescents or adults (by subtracting the maximum reported sexual attraction to either prepubertal girls or boys from the maximum self-reported sexual attraction to either of the four other categories [pubescent girls and boys, sexually mature men and women]). The teleiophilic subsample consisted solely of men indicating highest sexual attraction to adults (subtracting the maximum sexual attraction to either sexually mature men or women from the maximum sexual attraction to either of the four remaining stimuli classes). Those whose sexual attraction was highest for early-to-mid-pubertal adolescents or equally high for pubescent and prepubescent stimuli were categorized as the hebephilic subsample. Hence, the hebephilic group included seven individuals with an equally strong attraction to prepubescent and pubescent children. For the present analyses, the pedophilic and hebephilic group were combined into a pedohebephilic subsample. As categorization was based entirely on comparisons of self-reported sexual interests to different groups of people, a difference of at least one unit between the stimulus categories on the sexual interest scale was sufficient for categorization. Yet, one person in the teleiophilic group indicated a sexual interest in prepubescent children that surpassed the midpoint of the 10-point scale. Data from nine men who reported to have equally strong teleiophilic attraction and sexual attraction to one of the remaining four categories were excluded from the analyses.

We collected data from 89 self-reported teleiophilic men and 101 self-reported pedohebephilic men. Table 2 gives an overview of self-reported maximum sexual attraction ratings as a function of sexual interest groups and sexual maturity status. The pedohebephilic group was then split up into two subgroups according to their self-reported status regarding previous convictions for sexual offending (child pornography offending and/or child sexual abuse, see Table 2 for more information on the nature of the convictions). In the teleiophilic group, only one person self-reported a sexual offense involving children and was discarded from the analyses. The groups will be referred to as *Pedo-SO* (pedohebephilic men who do not report sexual offending), *Pedo* + *SO* (pedohebephilic men who do report sexual offending), and *Tel-SO* (teleiophilic men who do not report sexual offending) in results section. Hence, analyses based on sexual attraction and sexual offending status included 71 Pedo-SO, 30 Pedo + SO, and 88 Tel-SO.

**Table 2:** Self-reported maximum sexual attraction to female and male members of each category of sexual maturity, viewing time scores, and sexual offending status among the pedophilic, hebephilic, and teleiophilic group (Study 1, Study 2)

	Study 1						Study 2					
Variable	Pedophilia		Hebephilia^a^		Teleiophilia		Pedophilia		Hebephilia^a^		Teleiophilia	
	*M* (*SD*)	*n*	*M* (*SD*)	*n*	*M* (*SD*)	*M* (*SD*)	*M* (*SD*)	*n*	*M* (*SD*)	*n*	*M* (*SD*)	*n*
Self-reported attraction to prepubescents	9.57 (0.64)	53	6.00 (2.83)	48	1.35 (0.97)	89	9.74 (0.87)	137	6.77 (2.43)	141	1.30 (1.09)	317
Self-reported attraction to early–mid-pubescents	6.28 (2.05)	53	9.50 (0.83)	48	2.60 (1.95)	89	6.31 (2.31)	137	9.62 (0.76)	141	1.78 (1.71)	317
Self-reported attraction to adults	3.42 (2.48)	53	4.38 (2.45)	48	9.54 (1.02)	80	3.69 (2.46)	137	5.18 (2.54)	141	9.62 (0.91)	317
Viewing time score T1—T5	916 (970)	31	395 (769)	35	-685 (816)	84	-	-	-	-	-	-
Viewing time score T1,2,3- T4,5	-	-	-	-	-	-	355 (869)	91	162 (781)	90	-831 (902)	313
Prior convictions for sexual offenses combined (in %)	26.4	53	33.3	48	1.1	89	14.6	137	13.5	141	1.3	317
Prior convictions for child sexual abuse (in %)	7.5	53	16.7	48	0.0	89	6.6	137	7.1	141	0.9	317
Prior convictions for rape (in %)	0.0	53	0.0	48	0.0	89	2.2	137	0.7	141	0.0	317
Prior convictions for child pornography offenses (in %)	20.8	53	25.0	48	1.1	89	12.4	137	7.8	141	0.3	317

^a^Includes seven (Study 1) and 31 (Study 2) participants with an equally strong sexual attraction to prepubescents and early-to-mid-pubescents

#### Classification Based on Viewing Time

 For 42 participants, we could not record any response latencies likely due to their use of anonymity software which did not permit the task to run properly. Response latency outliers were screened for each single trial utilizing the *adjbox* function from the R package *robustbase* (Maechler et al., 2019). This procedure is based on a robust nonparametric modification of the standard Tukey criterion that specifically fits skewed distributions (Hubert & Vandervieren, 2008) which are notorious for response latency data. Empirically determined outliers were set to missing values (6% of the trials).

We calculated average response latencies for each Tanner by stimulus sex category, if we had recorded at least one value for each of the relevant stimulus categories (no cases were deleted because of outlier exclusion). We categorized groups based on the difference score between maximum response latencies to either male or female Tanner stage 5 and Tanner stage 1 stimuli. Similar difference coefficients have been shown to produce the most valid differentiation between teleiophilic and pedohebephilic groups (Schmidt et al., 2017). Participants with positive difference scores were classified as pedohebephilic subsample (although technically reflecting pedophilic preferences for prepubescent children), while participants with negative difference scores were coded as teleiophilic subsample. Based on their VT profiles, we were able to categorize 67 participants as pedohebephilic and 90 participants as teleiophilic. When considering offending status, this left us with 46 VT-based Pedo-SO, 21 Pedo + SO, and 86 Tel-SO.

#### Statistical Procedure for Main Analyses

Reverse Helmert contrasts were employed for planned comparisons to assess whether there were any differences between Pedo-SO, Pedo + SO, and Tel-SO. Specifically, we compared Tel-SO with the mean of Pedo-SO/ + SO and Pedo-SO with Pedo + SO. Because we had planned comparisons, we bypassed omnibus analysis of variance. For some analyses, we opted against the use of parametric tests because the distribution of residuals showed severe deviations from the normal distribution or because of unequal variances (heteroscedasticity). In these cases, we used appropriate robust tests (bootstrapping or Welch corrections).

## Results

### Agreement Between Self-Reported and Viewing Time-Inferred Sexual Interests

The two classification procedures led to similar results for 127/150 (85%) men who were sorted concordantly as either teleiophilic or pedohebephilic based on self-report and VT (χ^2^ = 70.98, *df* = 1, φ = 0.69, *p* < 0.001). Pedophilic and hebephilic men showed positive average scores (indicating stronger sexual attraction to children at Tanner stage 1 than adults at Tanner stage 5), while teleiophilic men achieved negative scores (indicating stronger attraction to adults than to children, see Table 2).

### Sample Description

Participants were rather well-educated (with 72%, 47%, and 82% reporting to have achieved University entrance certificates in Germany for Pedo-SO, Pedo + SO, and Tel-SO, respectively). Pedo + SO were significantly less educated than Tel-SO (χ^2^ = 13.99, *df* = 1, *p* = 0.001; ϕ = 0.34), while we detected no difference between Pedo-SO and Tel-SO (χ^2^ = 2.24, *df* = 1, *p* = 0.135; ϕ = 0.12). Pedo-SO/ + SO were older than Tel-SO, and Pedo + SO were older than Pedo-SO (both significant, Tables 3, 4). Sample descriptions based on VT-inferred classifications can be found in Supplement A.

**Table 3:** Planned comparisons (Helmert contrasts): Self-reported pedohebephilic men vs. teleiophilic men (Study 1)

Variable	Pedohebephilia (P –SO, P + SO)		Teleiophilia, no sexual offending (T-SO)		(P-SO, P + SO) vs. T-SO		
	*M* (*SD*)	*n*	*M* (*SD*)	*n*	*t (df)*	*p*	*d*^a^
Attraction to prepubescents	7.87 (2.68)	101	1.32 (0.93)	88	− 22.15^***^ (75.86)^b^	< .001	-3.20
Attraction to early–mid-pubescents	7.81 (2.26)	101	2.53 (1.87)	88	− 17.00^***^ (186)	< .001	− 2.54
Attraction to adults	3.87 (2.50)	101	9.55 (1.03)	88	18.76^***^ (65.62)^b^	< .001	2.92
Age	37.52 (12.44)	101	32.53 (11.45)	88	− 4.00 ^***^ (104.31)^b^	< .001	− 0.42
Viewing time score T1—T5	640 (901)	66	-692 (818)	83	− 9.46^***^ (146)	< .001	− 1.57
Height	180.54 (6.55)	101	180.55 (6.52)	88	0.21 (186)	.836	0.00
EHI Laterality Index	0.83 (0.48)	101	0.88 (0.43)	88	0.63 (186)	.535^c^	0.10
Head injuries before age 13	0.24 (0.67)	101	0.27 (0.64)	88	0.46 (186)	.619	0.05
Head injuries after age 13	0.20 (0.69)	101	0.31 (0.81)	87	0.69 (185)	.544^c^	0.14
Social desirability	1.97 (0.56)	100	1.86 (0.50)	88	-1.47 (185)	.144	− 0.20

^***^p < .001 (two-sided)

^a^*d* = *M*_1_–*M*_2_ / *SD*_pooled_, calculated using the *cohen.d* function of the R package *psych*

^b^We used Welch’s correction due to unequal variances (as indicated by Levene test for equality of variances, center = median)

^c^*p*-value based on 1,000 bootstrap samples due to severe deviations from the assumption that residuals are normally distributed

**Table 4:** Planned comparisons (Helmert contrasts): Self-reported pedohebephilic men with vs. without convictions for sexual offending (Study 1)

Variable	Pedohebephilia, no sexual offending (P –SO)		Pedohebephilia, sexual offending (P + SO)		P-SO vs. P + SO		
	*M* (*SD*)	*n*	*M* (*SD*)	*n*	*t (df)*	*p*	*d*^a^
Attraction to prepubescents	7.83 (2.77)	71	7.97 (2.48)	30	0.24 (60.60)^b^	.809	0.05
Attraction to early–mid-pubescents	7.63 (2.26)	71	8.23 (2.25)	30	1.32 (186)	.188	0.27
Attraction to adults	3.92 (2.44)	71	3.77 (2.67)	30	− 0.26 (50.34)^b^	.794	− 0.06
Age	34.03 (10.09)	71	45.80 (13.69)	30	4.25^***^ (42.9)^b^	< .001	1.06
Viewing time score T1—T5	552 (879)	43	805 (938)	23	1.15 (146)	.253	0.29
Height	180.85 (6.54)	71	179.83 (6.62)	30	− 0.71 (186)	.479	− 0.16
EHI Laterality Index	0.83 (0.49)	71	0.83 (0.46)	30	− 0.01 (186)	.994^c^	0.00
Head injuries before age 13	0.25 (0.73)	71	0.20 (0.48)	30	− 0.38 (186)	.652^c^	− 0.08
Head injuries after age 13	0.16 (0.58)	71	0.30 (0.92)	30	0.84 (185)	.463^c^	0.20
Social desirability	1.95 (0.55)	71	2.01 (0.60)	29	0.52 (185)	.601	0.11

^***^p < .001 (two-sided)

^a^*d* = *M*_1_–*M*_2_ / *SD*_pooled_, calculated using the *cohen.d* function of the R package *psych*

^b^We used Welch’s correction due to unequal variances (as indicated by Levene test for equality of variances, center = median)

^c^*p*-value based on 1,000 bootstrap samples due to severe deviations from the assumption that residuals are normally distributed

### Group Differences

Unless stated otherwise, results reported here refer to classification based on self-report. Readers can access results based on VT-based classification in the Supplemental Materials. We could not detect significant differences for any of the classification procedures (see Tables 3, 4 for self-report-based classification and Table S1-S2 in Supplement B for VT-based classification,), which was expected given the low statistical power. Therefore, we put a stronger focus on effect size point estimates than the significance of the effects.

Further data inspection revealed that the Pedo + SO group was about 1 cm shorter than the Pedo-SO and Tel-SO (see Table 3). Our results also suggested a higher likelihood for Pedo-SO/ + SO to be left-handed compared to Tel-SO (see Table 3), while there was no indication for differences in handedness depending on sexual offending status among the pedohebephilic group (see Table 4). Furthermore, our data indicated lower rates of head injuries before age 13 for Pedo-SO/ + SO compared to Tel-SO (see Table 3). Again on a descriptive level, Pedo + SO reported more head injuries after age 13 than Pedo-SO (Table 4). All three groups achieved similar scores on the social desirability scale (Tables 3, 4). In group comparisons based on VT-inferred classifications of sexual orientation, we achieved results in similar directions, with the sole difference that Pedo + SO were about 0.40 cm taller than Pedo-SO, and that Pedo-SO/ + SO appeared more likely to be right-handed than Tel-SO (all *ns*., see Tables S1—S2 in Supplement B).

### Control Analyses

As group differences in age might have biased our results, we assessed links between age, height, and head injuries before and after age 13 separately for each of the six tested subgroups (i.e., [1] self-reported Pedo-SO, Pedo + SO, and Tel-SO; [2] VT-inferred Pedo-SO, Pedo + SO, and Tel-SO). For the self-report classification, we found no significant link between age and height (Pedo-SO: *r* = .08, *p* = .482, 95%*CI* = [−.15, .31], Pedo + SO: *r* = −.33, *p* = .072, 95%*CI* = [−.62, .03], Tel-SO: *r* = −.13, *p* = .217, 95%*CI* = [−.33, .08]). Note that for head injuries, we determined significance based on 1,000 bootstrap samples because these variables showed severe deviations from the assumption of normality. There was no significant relationship between age and head injuries before age 13 (Pedo-SO: *r* = .07, 95%*CI* = [−.11, .40], Pedo + SO: *r* = .20, 95%*CI* = [−.25, .50], Tel-SO: *r* = .01, 95%*CI* = [−.19, .22]). We found, however, a significant negative relationship between age and head injuries after age 13 for non-offending teleiophilic participants (Pedo-SO: *r* = .18, 95%*CI* = [−.18, .47] Pedo + SO: *r* = .24, 95%*CI* = [−.16, 0.47], Tel-SO: *r* =  −.14, 95%*CI* = [−.25, -.003]). Similar non-effects were obtained for the VT-inferred classification, with the exception of a significant negative link between height and age among VT-based Pedo + SO (see Supplement C). Taking into account the number of tests, size, and direction of these effects, these observations speak against a systematic pattern for the head injuries measure. Nevertheless, they indicate, albeit not consistently, that differences in age may explain some of the (nonsignificant) differences for the contrasts based on height.

## Discussion

Study 1 showed that it is feasible—at least for a majority of the sampled individuals—to run an online version of the VT measure among self-identified males with sexual interest in children and adolescents. For the first time, these results extend the validation of VT tasks as measures of pedohebephilic sexual interest (Schmidt et al., 2017) to self-identified pedohebephilic men from the community which can be regarded as the most direct test of VT validity hitherto. Nevertheless, results of Study 1 are subject to typical caveats associated with research based on self-reported data, retrospective questionnaires, and online research (see general discussion for a detailed elaboration). In addition, statistical power was insufficient. Yet, descriptive analyses indicated that differences between pedohebephilic and non-pedohebephilic groups might be driven by a confound of (self-reported) sexual offending status. Furthermore, pedohebephilic and teleiophilic men reported similar rates of head injuries before age 13, irrespective of their offending status. Descriptively, our data appear to be more in line with research pointing toward a link between markers for neurodevelopmental differences and norm-breaking/criminal behavior, instead of pedohebephilia per se (with the exception of left-handedness, which was more prominent in the pedohebephilic group) calling for a more stringent test based on adequate statistical power. This was sought to achieve in Study 2.

## Study 2

### Method

#### Participants and Sample Size Calculations

To reach more potential participants, which is necessary to address the statistical power issue, and to make sure that samples in Study 1 and 2 are (at least largely) independent, Study 2 targeted English-speaking men. We conducted sample size calculations for all markers for neurodevelopmental differences based on effect sizes from the literature (see Table 1). We estimated that among all cognitive markers for neurodevelopmental differences, self-reported height would show the smallest average difference between pedohebephilic and teleiophilic participants. Hence, we set our sampling goal to 620 participants, based on estimations with G*Power (setting α = 0.05 and β = 0.20, one-sided testing for height, two-sided testing for all other hypotheses, Faul et al., 2007). The study was approved by the institutional review board of the MSH Medical School.

The study was advertised as a survey on wanted and unwanted childhood sexual experiences, cognitive development, and sexual interests in children or adults among men from the community. We collected data via the B4U-ACT support group for people with pedohebephilia between July 2018 and March 2019, offering to donate 1.50$ for each participant to B4U-ACT (with a maximum donation sum of $300). We furthermore recruited participants from the MTurk workforce. Only male MTurk workers who had been approved for 100 to 5000 human intelligence tasks with an overall approval rate of 80% or more were eligible for the study and received a payment of two US$. The following countries of residences were allowed for participation (task language was English): Australia, Austria, Belgium, Canada, Czech Republic, Denmark, Estonia, Finland, France, Germany, Greece, Iceland, Italy, Lithuania, Luxembourg, Netherlands, New Zealand, Norway, Poland, Portugal, Slovakia, Spain, Sweden, UK, and the USA. In sum, we recruited 331 participants via B4U-ACT and 318 via MTurk. After the exclusion of 17 people who did not disclose their sex or reported to be female or other, this left us with a total of 632 participants. Detailed information on how we categorized participants based on their sexual interests and offending status is presented in a separate section after the description of our measures.

#### Measures

Measurements were administered in the same order as they appear in the following section. Additionally, we assessed information about traumatic childhood experiences and non-negative sexual experiences in childhood for a separate publication (Jahnke et al., 2021).

##### Viewing Time Measure of Sexual Interest

We employed a similar VT task as in Study 1, again using stimulus material from the Not Real People set (Pacific Psychological Assessment Cooperation, 2004). Deviating from Study 1, we added pictures of early (Tanner stages 2–3) and late adolescents (Tanner stage 4), alongside pictures of adults (Tanner stage 5) and prepubescent children (Tanner stage 1). To shorten test duration, we presented only four pictures per sex and Tanner stage category.

##### Self-Reported Sexual Interests

 We used the English version of the measure from Study 1.

##### International Cognitive Ability Resource Sample Test

 To measure cognitive ability, we used the 16-item sample test from the public-domain International Cognitive Ability Resource (ICAR; Condon & Revelle, 2014), which consists of letter and number series, matrix and verbal reasoning items, and three-dimensional rotation items. Previous research has established that the ICAR 16-item sample test is a reliable and valid measure of global cognitive ability (Condon & Revelle, 2015; Merz et al., 2020). The (self-administered) ICAR-16 shows high convergent validity with the Wechsler Adult Intelligence Scale (fourth edition) administered by a trained clinician (*r* = 0.81 and *r* = 0.94 with the manifest and the latent score of the WAIS-IV, respectively, Young & Keith, 2020). As pedohebephilic participants might have a stronger motivation than teleiophilic MTurkers to excel on the IQ test, it was planned to compare their responses with scores obtained among Internet users who wanted to test their cognitive abilities in a previous study (Condon & Revelle, 2015). To match procedures employed in this comparison study, we presented the test without time restraints. For 18 participants (all recruited via B4U-ACT) we could not calculate sum scales due to missing values. Internal consistency was good (Kruder-Richardson 20 = 0.84).

##### Adapted Edinburgh Handedness Inventory

 We used the English version of the EHI (Dyshniku et al., 2015) employed in Study 1, again without the broom and box items (α = 0.89, no missing values).

##### Head Injuries

As in Study 1, we used items from Blanchard et al. (2013) to assess head injuries involving unconsciousness before and after age 13. One participant recruited via B4U-ACT left these questions unanswered.

##### Sociodemographic Information (Including Height and Previous Convictions)

As in Study 1, we assessed sex, age, and educational achievement (i.e., having or not having obtained a Bachelor's degree or higher). Participants were allowed to choose whether to report their height in the metric (cm) or the imperial system (the latter were subsequently transferred to cm). For height, we corrected obvious typing mistakes in four cases (e.g., 88 inches were changed to 8 inches). To determine outliers, we estimated fences based on the Tukey criterion (1.5 interquartile ranges). This led to the exclusion of five data points above 198.12 cm and six below 157.48 cm. Cases with heights outside the fences were about equally split between participants from MTurk (2 above, 3 below) and B4U-ACT (3 above, 3 below). Twelve participants from B4U-ACT and two from MTurk did not report their height. For the assessment of previous convictions, we administered the same items as in Study 1.

#### Categorizing Participants’ Sexual Interests and Offending Status

##### Classification Based on Self-Reported Sexual Attraction

 We formed groups based on self-reported sexual interests following procedures in Study 1. This led us to categorize 317 participants as teleiophilic men and 278 as pedohebephilic men (141 with a predominant pedophilic interest). Twenty-one participants from the self-reported teleiophilic group were recruited via B4U-ACT, while seven participants from the self-reported pedohebephilic group were sampled on MTurk. Those participants were not excluded from the analyses. Thirty-seven participants who reported equal maximum sexual attraction to adult and prepubertal/early-to-mid-pubertal persons were excluded. Among self-reported teleiophilic participants, only seven (2%) reported a sexual interest in prepubescent girls or boys surpassing the midpoint of the sexual attraction scale. Regarding girls or boys in early-to-mid-puberty, only 20 (6%) self-reported teleiophilic men reported sexual interests surpassing the midpoint of the respective scales. Group means for sexual attraction ratings are displayed in Table 2. In the teleiophilic group, four participants self-reported a sexual offense and were therefore discarded from the T-SO group. Analyses based on sexual attraction and sexual offending status included 239 Pedo-SO, 39 Pedo + SO, and 313 Tel-SO.

##### Classification Based on Viewing Time

 For 110 participants, we could not record any response latency data, most likely due to the use of anonymity software. Using the same method to identify outliers as in Study 1, 4% of recorded trials were marked as outliers. All participants had to have at least one valid response for each Tanner stage by stimulus sex category (note that this requirement was fulfilled in all cases where VT scores were recorded). Viewing time data were analyzed in the same way as described in Study 1, with one exception: As Study 2 included pictures from Tanner stages 1 to 5, we additionally calculated difference scores between maximum average response latencies to either male and female stimuli separately for each of the five Tanner stages. To determine pedohebephilia, we subtracted maximum average reaction times to either male or female stimuli in Tanner stages 4 and 5 (late pubescence and adulthood) from Tanner stages 1, 2, and 3 (pre-peri-pubescence). Participants with positive difference scores were classified as pedohebephilic, while participants with negative difference scores were classified as teleiophilic. Thus, we categorized 60 participants as pedophilic (i.e., having higher scores for Tanner stage 1 than for Tanner stages 2 to 5), 119 as hebephilic (i.e., having higher values for Tanner stages 2 and 3 than for Tanner stages 1, 4, and 5), and 326 as teleiophilic (i.e., having higher values for Tanner stages 4 and 5 than for Tanner stages 1 to 3). When accounting for offending status previous convictions for sexual offending (child pornography offending, rape, and/or child sexual abuse, see Table 2 for further information), 154 were categorized as Pedo-SO, 25 as Pedo + SO, and 326 as Tel-SO.

## Results

### Agreement Between Self-Reported and Viewing Time-Inferred Sexual Interests

 For the 493 participants that we could classify based on self-reported and VT-inferred sexual interests, classification procedures led to similar results (χ^2^ = 123.32, *df* = 1, φ = 0.50, *p* < 0.001). Of the self-reported teleiophilic participants, 267 (85%) showed a teleiophilic pattern in the VT task, while 114 (63%) of self-reported pedohebephilic participants showed VT results indicating pedohebephilic interests. The pedophilic and hebephilic groups achieved on average positive VT mean scores (indicating a stronger attraction to pedohebephilic stimuli on Tanner stage 1–3 than to late/postpubescent stimuli on Tanner stage 4 or 5), while the self-reported teleiophilic group showed negative VT scores (indicating a stronger relative attraction to physically mature adults, see Table 2).

### Sample Description

Educational levels were relatively high as 53%, 49%, and 62% indicated to have achieved an associate degree, BA degree, or higher among Pedo-SO, Pedo + SO, and Tel-SO, respectively. We detected no significant difference between Tel-SO, Pedo-SO, and Pedo + SO regarding educational level (χ^2^ = 5.27, *df* = 2, φ = 0.09, *p* = 0.072). Sexual offenses were reported mostly by pedohebephilic participants (Table 2). Age did not differ significantly between Pedo-SO/ + SO and Tel-SO, but Pedo + SO were significantly older than Pedo-SO (Tables 5–6). Sample descriptions based on VT are presented in Supplementary Material A.

**Table 5:** Planned comparisons (Helmert contrasts): Self-reported pedohebephilic men vs. teleiophilic men (Study 2)

Variable	Pedohebephilia (P –SO, P + SO)		Teleiophilia, no sexual offending (T-SO)		(P-SO, P + SO) vs. T-SO		
	*M* (*SD*)	*n*	*M* (*SD*)	*n*	*t (df)*	*p*	*d*^a^
Attraction to prepubescents	8.23 (2.36)	278	1.29 (1.09)	313	− 38.97^***^ (75.36)^b^	< .001	− 3.87
Attraction to early–mid-pubescents	7.99 (2.38)	278	1.77 (1.71)	313	− 30.31^***^ (92.57)^b^	< .001	− 3.04
Attraction to adults	4.44 (2.61)	278	9.64 (0.91)	313	23.15^***^ (53.09)^b^	< .001	2.73
Age	34.44 (12.98)	273	35.15 (11.06)	313	− 1.21 (113.35)^b^	.228	0.06
Viewing time score T1,2,3—T4,5	259 (830)	181	− 839 (904)	309	− 10.77^***^ (487)	< .001	− 1.25
Height	178.89 (7.55)	259	177.66 (7.18)	307	0.11 (563)	.545^c^	− 0.17
EHI Laterality Index	0.68 (0.61)	278	0.68 (0.58)	313	− 0.77^***^ (588)	.387^d^	0.00
ICAR	9.86 (4.18)	260	7.24 (3.52)	313	− 3.23^**^ (78.43)^b^	.002	− 0.69
Head injuries before age 13	0.25 (0.66)	277	0.25 (0.68)	313	− 0.20 (587)	.867^d^	0.00
Head injuries after age 13	0.20 (0.64)	276	0.30 (0.78)	313	0.01 (64.35)^b^	.989^d^	0.14

^***^p < .001, ^**^p < .01 (two-sided)

^a^*d* = *M*_1_–*M*_2_ / *SD*_pooled_, calculated using the *cohen.d* function of the R package *psych*

^b^We used Welch’s correction due to unequal variances (as indicated by Levene test for equality of variances, center = median)

^c^One-sided *p*

^d^*p*-value based on 1,000 bootstrap samples due to severe deviations from the assumption that residuals are normally distributed

**Table 6:** Planned comparisons (Helmert contrasts): Self-reported pedohebephilic men with vs. without convictions for sexual offending (Study 2)

Variable	Pedohebephilia, no sexual offending (P –SO)		Pedohebephilia, sexual offending (P + SO)		P-SO vs. P + SO		
	*M* (*SD*)	*n*	*M* (*SD*)	*n*	*t (df)*	*p*	*d*^a^
Attraction to prepubescents	8.16 (2.41)	239	8.69 (1.92)	39	1.55 (59.48)^b^	.128	0.23
Attraction to early–mid-pubescents	7.98 (2.44)	239	8.03 (2.05)	39	0.12 (57.13)^b^	.908	0.02
Attraction to adults	4.63 (2.53)	239	3.31 (2.81)	39	− 2.76^**^ (48.56)^b^	.008	− 0.52
Age	33.59 (13.14)	234	39.51 (10.84)	39	3.06^**^ (58.31)^b^	.003	0.46
Viewing time score T1,2,3—T4,5	258 (860)	152	262 (659)	29	0.02 (487)	.982	0.00
Height	179.38 (7.34)	224	175.77 (8.18)	35	− 2.72^**^ (563)	.007	− 0.49
EHI Laterality Index	0.66 (0.62)	239	0.79 (0.54)	39	1.27 (588)	.167^c^	0.21
ICAR	10.35 (4.00)	224	6.78 (4.02)	36	− 4.95^***^ (46.81)^b^	< .001	− 0.90
Head injuries before age 13	0.24 (0.61)	239	0.29 (0.90)	38	0.40 (587)	.763^c^	0.07
Head injuries after age 13	0.16 (0.56)	239	0.43 (0.99)	37	1.64 (39.63)^b^	.131^c^	0.44

^***^p < .001, ^**^p < .01 (two-sided)

^a^*d* = *M*_1_–*M*_2_ / *SD*_pooled_, calculated using the *cohen.d* function of the R package *psych*

^b^We used Welch’s correction due to unequal variances (as indicated by Levene test for equality of variances, center = median)

^c^*p*-value based on 1,000 bootstrap samples due to severe deviations from the assumption that residuals are normally distributed

### Group Differences

 For the self-report-based classification, we found no differences between Pedo-SO/ + SO and Tel-SO regarding height, laterality index, and head injuries before and after age 13 (Table 5). Yet, we detected significant differences in IQ scores, with Pedo-SO/ + SO achieving higher scores than Tel-SO. P-SO also emerged as significantly taller and more intelligent than P + SO (Table 6). Furthermore, unobtrusively recorded response latencies during the IQ test revealed that the Pedo-SO group had an almost twice as long average completion time as the Pedo + SO and the Tel-SO group (with median scores of 15.09 min compared to 7.65 min and 8.40 min, based on reaction time data from 153 Pedo-SO, 29 Pedo + SO, and 310 Tel-SO).^2^ To compare results with a sample that was arguably more motivated to score high, we used data from the ICAR project (Condon & Revelle, 2015) to extract means and standard deviations for each of the items from the ICAR 16-item sample test among men over 18 years (*n* between 4220 and 13,216, average *n* = 9324, note that the ICAR items were delivered untimed in both our survey and the ICAR project). All of these participants had solved a varying number of the ICAR item set online in order to receive a personalized feedback (Condon & Revelle, 2014). These individual means and standard deviations were added to create average sum scores (*M* = 8.60, *SD* = 7.42). *t*-tests with Welch-adjusted degrees of freedom based on these summary statistics revealed that Pedo-SO/ + SO (*M* = 9.86, *SD* = 4.18, *n* = 260) scored higher than participants from the ICAR project. Yet, albeit significant (*t*(306.45) = 4.66, *p* < 0.001, *d* = 0.21), effect sizes were smaller than the ones we retrieved for the comparison with the Tel-SO within the current study.

When the classification of sexual attraction was based on VT, none of the contrasts for markers for neurodevelopmental differences reached significance (see Supplementary Material Tables S3–S4). Note that sample sizes were smaller in the alternative classification, so failure to reach significance was more likely. Yet, differences descriptively tended to be similar in magnitude and direction, with the exception of Pedo-SO/ + SO showing a stronger preference for the right hand compared to Tel-SO.

### Control Analyses

To determine whether age could be a confound, we assessed its link to IQ, height, and head injuries before and after age 13 separately for each of the six tested subgroups (i.e., [1] self-reported Pedo-SO, Pedo + SO, and Tel-SO; [2] VT-inferred Pedo-SO, Pedo + SO, and Tel-SO). For variables that deviated severely from the assumption of normality (in this case head injuries before or after 13), we used bootstrapping procedures. For the self-report based classification, only 2/12 tests showed a significant link between older age and any of the dependent variables (head injuries before age 13 and ICAR among Pedo-SO, *r* = 0.13, 95%*CI* based on bootstrap samples = [0.008, 0.29] and *r* = 0.14, *p* = 0.033, 95%*CI* = [0.01, 0.27], respectively). For the VT-based classification, we could also only detect significant results for 1/12 tests (see Supplemental Material C). Considering the large number of tests and the direction of the effects, the evidence for age confounds was weak, as the number of significant tests does not fall far from the margin of what would be expected by chance at the given significance threshold of *p* < 0.05.

## General Discussion

We could not corroborate our first hypothesis that pedohebephilic men from our two community samples differed significantly from teleiophilic men with respect to height, intelligence, non-right-handedness, or rate of head injuries during childhood. We did, however, find evidence for the second hypothesis that pedohebephilic men who have sexually offended were smaller and less intelligent than non-offending pedohebephilic men. Replicating Gerwinn et al.'s (2019) findings, the current studies do not yield support for the theory that sexual interests in children among community men are linked to neurodevelopmental differences. Yet, in contrast to Gerwinn et al. (2019), which suffered from lacking statistical power, Study 2 was able to test differences between pedohebephilic and teleiophilic men at least 1-β = 0.80, with the exception of height.

Because of the limitations of the present case–control design and the impossibility to prove a null hypothesis in classical hypothesis testing, our findings cannot conclusively disprove the idea of a general link between indicators of neurodevelopmental perturbations and pedohebephilia. Nevertheless, the present data are positively commensurate with the notion that indicators of neurodevelopmental perturbations such as, particularly, height and intelligence are linked to (self-reported) offending status when pedophilic interest is kept constant in group comparisons. This dovetails with the fact that all markers of neurodevelopmental perturbations as assessed here are empirical correlates of criminal behavior—as outlined in introduction (see also Beckley et al., 2014; Bogaert, 2001; McKinlay et al., 2014; Moffitt et al., 1994). Future research is needed to clarify if the importance of neurobiological alterations might have been overstated in the previous literature, as part of the variance attributed to pedohebephilia may in fact have to be attributed to other factors due to the lack of specificity of the neurodevelopmental perturbations hypothesis.

### Limitations and Outlook

Our results are subject to a number of caveats, some of which are specific to our setting and methodology, while others are generally associated with research on proxy measures of neurodevelopmental perturbations. First and foremost, correlational analyses are unfit to prove causation—a fact that applies to most studies in the field of the neurodevelopmental perturbations hypothesis and that increases the risk of falling victim to third variable confounds in case–control study designs.

Additionally, our study populations are neither representative for teleiophilic nor pedohebephilic men. While clinical, institutional, or forensic samples are subject to selection biases as well, this means that results have to be interpreted carefully with respect to confounds and (self-selection) biases. It is, for instance, possible that tall pedohebephilic men with high levels of cognitive functioning were more likely to participate than those with lower height or lower levels of cognitive abilities, given that they were aware of hypotheses from the academic literature. Yet, as online studies generally tend to oversample younger and more educated participants, this limitation also applies to the teleiophilic groups. It is also possible that links between markers of neurodevelopmental perturbations and pedohebephilia exist in the population but cannot be identified when assessing subgroups with high computer literacy. Even if pedohebephilia was linked to such markers, it may be impossible to detect these links in samples without individuals with low levels of cognitive functioning. Yet, note that in forensic settings, pedohebephilic men who have been convicted for child pornography offending (i.e., indicating at least average levels of computer literacy and cognitive capacity) showed more markers of neurodevelopmental perturbations than teleiophilic men who have committed the same crime (Blanchard et al., 2007). In the absence of representative samples of teleiophilic and pedophilic men, it is impossible to determine if our results are due to such ceiling effects.

Moreover, participants from the pedohebephilia groups were recruited via forums addressing sexual attraction to children, where they may have been looking for support because they experienced increased levels of distress. Indeed, previous research indicates that pedohebephilic participants in online surveys generally report higher levels of distress than population-based or other non-clinical control samples. Yet, it is also shown that they are considerably less distressed than self-referred pedohebephilic patients in treatment projects (Jahnke et al., 2015).

The generalizability of the present findings is further constrained by the WEIRDness (Western, Educated, Industrialized, Rich, Democratic; Henrich et al., 2010) of the present samples. Note also that we cannot guarantee that Study 1 and 2, without exception draw from two completely independent participant pools. It is possible that some participants are fluent in both German and English, have visited both German and English language web forums for pedohebephilic people, and have participated in both studies. Future research drawing on online samples of pedohebephilic men should include an item to identify and potentially screen out participants from previous studies on the neurodevelopmental markers of pedohebephilia.

Furthermore, both studies only included relatively small numbers of pedohebephilic men who reported convictions for sexual offenses. This means that even though some contrasts yielded significant results, small changes in participant characteristics can lead to fluctuations in the results. Although difficult to attain, replications with larger samples of men with sexual interests in children who have sexually offended are needed to corroborate these effects with more certainty. Preferably, these should be men from the community to control for selection factors related to being institutionalized in forensic settings.

Additionally, readers need to keep in mind that offending status was determined based on self-reported convictions for rape, child sexual abuse, and/or child pornography offenses alone. Hence, there may be people in our non-offending sample who have sexually offended but were not detected or convicted, which makes it impossible to tell whether effects are a function of criminal sexual behavior or of being convicted for such acts. Previous research also indicates that men convicted for child pornography offenses may differ from men convicted for child sexual offending on a number of relevant variables, such as antisociality (Babchishin et al., 2015). Hence, it is possible that we would have found larger differences between the groups with and without a history of sexual offending, if we had had a higher rate of pedohebephilic men convicted for child sexual offending compared to child pornography offending. Yet, note that a study comparing pedohebephilic people with either of these convictions shows more similarities than differences between the groups (Neutze et al., 2011). Furthermore, because we did not assess previous non-sexual convictions, we could not screen out people with a significant non-sexual offense history as an indicator of antisociality. This is a potential limitation, as antisociality may be linked to neurodevelopmental perturbations. Yet, given the nature of our surveys and previous research among MTurk samples, our samples are unlikely to include people with marked antisocial traits.

Another concern is that pedohebephilic participants might have been more motivated to perform optimally in order to better the image of their group. These motivational differences became apparent in the intelligence measure in Study 2, which involved complex puzzles that required effort to solve. Teleiophilic MTurk workers, who purportedly had less to gain from putting extra efforts into the cognitive tests, spent much less time on solving the items than the people from the pedohebephilic group. To get a perhaps more accurate estimation of pedohebephilic men's cognitive abilities, we additionally compared responses from our pedohebephilic sample in Study 2 with test data from Condon and Revelle's (2014) validity study on the ICAR items. As participants in their dataset had been recruited online with the prospect to receive customized feedback, we expected that these participants had put more effort into solving the presented logical puzzles compared to our teleiophilic sample. Yet, while these participants scored higher on the test items than teleiophilic men from Study 2, pedohebephilic men still emerged as more intelligent.

One of the main drawbacks of online research is the lack of control that researchers can exert on the setting. In contrast to laboratory surveys, we were not able to make sure that participants stayed focused during the IQ test or correctly followed all instructions, and doubts remain whether IQ tests in non-controlled online environments compare with clinician administered IQ inventories. Yet, note that previous research found no structural differences regarding matrices intelligence scores obtained in online and offline samples (Ihme et al., 2009). Notably, although the presence of an interviewer can be advantageous, it may also introduce an expectancy bias on the interviewer’s side. For instance, clinical staff may look harder for anomalies when examining pedophilic men, or they may more readily accept a teleiophilic man's self-report to have never had an accident leading to unconsciousness, prompting them to ask fewer follow-up questions. Reporting biases due to perceived demand effects on the interviewee’s side (and/or the larger clinical-forensic context) are also conceivable. In the present studies, where pedophilic and non-pedophilic men were prompted with the same set of questions, such types of bias are unlikely.

We were also limited in the range of variables we were able to assess, as many potentially interesting outcome variables are difficult (e.g., minor physical anomalies or 2D:4D digit ratio; Jordan et al., 2020) or even impossible (e.g., brain morphology and functioning; Cantor et al., 2008; Massau et al., 2017; Schiffer et al., 2017) to measure in an online setting. For obvious reasons, this precludes the assessment of large numbers of pedohebephilic men without a history of sexual offending as a mostly hidden and hard-to-reach population. On the one hand, this limits our ability to judge the validity of the neurodevelopmental perturbations theory in its entirety, as the evidence for this hypothesis goes beyond the factors examined here.

Furthermore, with exception of the VT measure and the IQ test, the online environment forced us to rely completely on self-report, which puts restraints on the validity of the data assessed. The tendency of men to over-report their height is well-documented (Gorber et al., 2007), and pedophilic and teleiophilic men who have committed sexual offenses are no exception to this rule: Fazio et al. (2014) report differences between actual and self-reported height varying between 2 and 4 cm, similar to differences observed in non-clinical samples (e.g., Palta et al., 1982). This means that the data we obtained on height in both our pedophilic and teleiophilic samples are likely to be biased. However, this is potentially a common limitation, at least for earlier studies, which, astonishingly, either report to have relied on self-reported height (e.g., Cantor et al., 2007) or had no access to information on how height was assessed (Levenson & Ackerman, 2017; but note that this is less likely to be the fact for newer research, Fazio et al., 2017). Despite those problems, online research may represent the only feasible way of reaching participants with pedohebephilia (as a dominant sexual attraction) outside of clinical or forensic contexts.

The present paper only focused on how men with pedohebephilic (who have and have not sexually offended) and teleiophilic interests differed in terms of neurodevelopmental indicators. Future studies based on larger samples than the present one should consider conducting additional more fine-grained analyses (e.g., to differentiate between pedophilic, hebephilic, and teleiophilic participants or between different types of sexual offenses involving children).^3^ It also needs to be stressed that, following conventions in the field, the present study was conducted with a type II error rate of 0.20, meaning that there is a 20% chance that the null hypothesis (i.e., that there is no difference between pedohebephilic and teleiophilic men) is falsely accepted.

### Conclusions

Our findings underscore a general conclusiveness problem arising from case–control designs: The interpretation of findings is largely dependent on the composition of the comparison groups. While there is no such thing as a representative sample of men with pedohebephilic interests, studies from community settings represent an important corrective to data from pedohebephilic men "who are available for study because they are either distressed by their sexual interests […] or criminally charged for sexual offenses" (Seto, 2004, p. 323). Whatever processes may ultimately be involved in the development of pedohebephilic interests, evidence from recent surveys involving non-offending pedohebephilic individuals indicates that the claim that pedohebephilia is based on neurodevelopmental perturbations might not be as inevitable and generalizable as previously thought by bearing the potential to disentangle “institutional caseness,” criminality, and sexual interest (Gerwinn et al., 2018). Moreover, as the neurodevelopmental perturbation account is silent on the actual processes that are involved in developing pedohebephilia (and which thus have never been subjected to empirical testing) only proxy measures that itself are just correlates of the supposed developmental trajectory have hitherto been examined. The potential of such correlates (i.e., the perturbation proxy measures used here) of correlates (i.e., one process among possibly many etiological pathways) to be confounded with third variables is high and leads to substantial methodological problems in gathering empirical support for the theory—specifically if it refers to a phenomenon that is linked to criminal behavior, publicly despised (Lehmann et al., 2020), and has a low base-rate (Bártová et al., 2021; Dombert et al., 2016).

Last but not least, it is noteworthy that data from two samples corroborated the validity of VT assessments in self-identified community males with pedohebephilic sexual interests by yielding theoretically meaningful differences in a known-groups approach. This can be regarded as the hitherto most accurate estimator of the validity of VT measures, as prior studies rested on various group comparisons with men convicted for child sexual abuse. The latter groups, however, are to a large degree only a pedohebephilia proxy as they are composed of pedohebephilic and teleiophilic individuals thus yielding only a conservative (i.e., lower-bound) estimator of the magnitude of possible group differences (Schmidt et al., 2017).

## Supplementary Information

Below is the link to the electronic supplementary material.

Supplementary file1 (PDF 120 KB)

## Footnotes

1 The large majority (63%) of the participants scored between 10 and 12 points on the crystallized intelligence test (note that the total score could theoretically range from 0 [no correct answer] to 12 [all items answered correctly]), indicating ceiling effects. Furthermore, a sizeable number of participants correctly recalled even 12-digit numbers on the working memory test, which raised doubts whether the digit span trials were performed according to instructions.

2 Pedo-SO/ + SO had higher scores on the ICAR test than Tel-SO, even when controlling for completion time via an ANCOVA design (*F*(1, 489) = 25.25, *p* < .001). Yet, note that although ANCOVAs represent a common strategy to "control" for confounds, we have doubts that this strategy is appropriate and leads to meaningful conclusions (see Miller & Chapman, 2001 for a non-technical discussion of common misuses of ANCOVA in psychopathology research).

3 The authors are willing to share the results of such more detailed analyses based on our datasets (e.g., for the purpose of meta-analysis) upon request.
