# Supplementary material for: Neurodevelopmental Differences, Pedohebephilia, and Sexual Offending: Findings from Two Online Surveys
Source: Arch Sex Behav. 2022 Jan 6;51(2):849–66. doi: 10.1007/s10508-021-02228-w (PMC8888371; doi:10.1007/s10508-021-02228-w)
Supplement: Supplementary file 1 — Supplementary file1 (PDF 120 KB) [file 10508_2021_2228_MOESM1_ESM.pdf]

*Supplementary Material A: Sample Descriptions based on Viewing Time*

## Study 1

With 83% reporting to have achieved the Abitur, VT-inferred teleiophilic men were more likely to be highly educated than VT-inferred pedohebephilic men, who had Abitur in 61% of cases ($\chi^2 = 9.76$, $df = 1$, $p = .002$, $\varphi = .25$). In the pedohebephilic group, 11.9%, 0.0%, and 23.9% reported prior convictions for child sexual abuse, rape, and child pornography offenses, respectively. In the teleiophilic group, 4.4% reported prior convictions for child pornography offenses, but none reported convictions for child sexual abuse or rape. Pedo-SO/+SO were older than Tel-SO, and Pedo-SO were younger than Pedo+SO (see Table S1 and S2 in Supplement B). There were no differences in educational level between Pedo-SO and Pedo+SO ($\chi^2 = 2.37$, $df = 1$, $\varphi = -.19$, $p = .123$), but between Pedo-SO/+SO and Tel-SO ($\chi^2 = 9.90$, $df = 1$, $\varphi = .25$, $p = .002$)

## Study 2

We found high education levels in the three VT-based groups (42%, 60%, and 59% with an associate degree, BA. degree, or higher in VT-based pedophilic, hebephilic, and teleiophilic group, respectively). Among the three groups, 18%, 14%, and 4% reported convictions for sexual offenses (for pedophilic, hebephilic, and teleiophilic men, respectively). Differences between Pedo-SO, Pedo+SO, and Tel-SO were not significant regarding educational level ($\chi^2 = 4.26$, $df = 2$, $\varphi = .01$, $p = .118$) or age (see Table S3).

*Supplementary Material B: Results for classification based on viewing time.*

Table S1. Planned comparisons (Helmert contrasts): VT-based pedohebephilic men vs. teleiophilic men (Study 1)

| Variable | Pedohebephilia (P –SO, P+SO) | | Teleiophilia, no sexual offending (T-SO) | | (P-SO, P+SO) vs. T-SO | | |
|---|---|---|---|---|---|---|---|
| | *M* (*SD*) | *N* | *M* (*SD*) | *n* | *t (df)* | *p* | *d* [a] |
| VT T1 | 2543 (857) | 67 | 1550 (538) | 86 | -8.29*** (66.93)[b] | <.001 | -1.44 |
| VT T5 | 1660 (560) | 67 | 2382 (767) | 86 | 6.11*** (150) | <.001 | 1.06 |
| Age | 38.81 (14.58) | 67 | 32.02 (11.61) | 86 | -3.79*** (64.26) [b] | <.001 | -0.53 |
| Height | 179.33 (7.26) | 67 | 180.59 (6.58) | 86 | 1.01 (150) | .315 | 0.18 |
| EHI Laterality Index | 0.88 (0.36) | 67 | 0.84 (0.49) | 86 | -0.67 (150) | .505 | -0.09 |
| Head Injuries before age 13 | 0.16 (0.48) | 67 | 0.36 (0.82) | 86 | 1.49 (150) | .135 [c] | 0.28 |
| Head Injuries after age 13 | 0.26 (0.77) | 67 | 0.32 (0.85) | 85 | 0.08 (149) | .936 [c] | 0.07 |
| Social Desirability | 1.99 (0.59) | 66 | 1.90 (0.52) | 86 | -1.20 (149) | .230 | -0.17 |

*** $p < .001$ (two-sided)

[a] $d = M_1 – M_2 / SD_{pooled}$, calculated using the *cohen.d* function of the R package *psych*

[b] we used Welch's correction due to unequal variances (as indicated by Levene test for equality, center = median)

[c] *p*-value based on 1,000 bootstrapped samples due to severe deviations from the assumption that residuals are normally distributed

Table S2. Planned comparisons (Helmert contrasts): VT-based pedohebephilic men with vs. without convictions for sexual offending (Study 1)

| Variable | Pedohebephilia, no sexual offending (P –SO) | | Pedohebephilia, sexual offending (p+SO) | | P-SO vs. P+SO | | |
|---|---|---|---|---|---|---|---|
| | *M* (*SD*) | *N* | *M* (*SD*) | *n* | *t (df)* | *p* | *d* [a] |
| VT T1 | 2488 (883) | 46 | 2665 (805) | 21 | 0.81 (42.33) [b] | .423 | 0.21 |
| VT T5 | 1647 (565) | 46 | 1687 (562) | 21 | 0.22 (150) | .827 | 0.07 |
| Age | 35.57 (13.18) | 46 | 45.90 (15.3) | 21 | 2.68* (34.10) [b] | .011 | 0.76 |
| Height | 179.20 (7.47) | 46 | 179.62 (6.94) | 21 | 0.23 (150) | .816 | 0.06 |
| EHI Laterality Index | 0.87 (0.38) | 46 | 0.92 (0.33) | 21 | 0.41 (150) | .685 | 0.13 |
| Head Injuries before age 13 | 0.13 (0.45) | 46 | 0.24 (0.54) | 21 | 0.59 (150) | .435 [c] | 0.23 |
| Head Injuries after age 13 | 0.18 (0.57) | 46 | 0.43 (1.08) | 21 | 1.14 (149) | .323 [c] | 0.32 |
| Social Desirability | 1.96 (0.56) | 46 | 2.06 (0.67) | 20 | 0.68 (149) | .496 | 0.17 |

* $p < .05$ (two-sided)

[a] $d = M_1 – M_2 / SD_{pooled}$, calculated using the *cohen.d* function of the R package *psych*

[b] we used Welch's correction due to unequal variances (as indicated by Levene test for equality of variances, center = median)

[c] *p*-value based on 1,000 bootstrapped samples due to severe deviations from the assumption that residuals are normally distributed

Table S3. Planned comparisons (Helmert contrasts): VT-based pedohebephilic men vs. teleiophilic men (Study 2)

| Variable | Pedohebephilia (P –SO, P+SO) | | Teleiophilia, no sexual offending (T-SO) | | (P-SO, P+SO) vs. T-SO | | |
|---|---|---|---|---|---|---|---|
| | *M* (*SD*) | *N* | *M* (*SD*) | *n* | *t (df)* | *p* | *d* [a] |
| VT T1 | 2160 (865) | 179 | 1419 (640) | 326 | -9.70*** (69.81) [b] | <.001 | -1.07 |
| VT T2, 3 | 2544 (794) | 179 | 1658 (670) | 326 | -8.86*** (43.55) [b] | <.001 | 0.75 |
| VT T4, 5 | 2096 (695) | 179 | 2705 (999) | 326 | 6.91*** (85.69) [b] | <.001 | 0.26 |
| Age | 34.38 (12.39) | 179 | 35.15 (11.89) | 326 | -1.90 (502) | .058 | -0.07 |
| Height | 178.04 (7.47) | 171 | 178.27 (7.32) | 317 | 0.69 (485) | .491 | -0.06 |
| EHI Laterality Index | 0.75 (0.54) | 179 | 0.67 (0.59) | 326 | -1.83 (502) | .068 | 0.02 |
| ICAR | 8.79 (4.04) | 179 | 7.52 (3.69) | 326 | -1.75 (46.36) [b] | .086 | 0.16 |
| Head Injuries before age 13 | 0.30 (0.63) | 179 | 0.26 (0.70) | 326 | -0.98 (501) | .387 [c] | 0.27 |
| Head Injuries after age 13 | 0.20 (0.61) | 178 | 0.30 (0.80) | 326 | 0.12 (501) | .929 [c] | 0.47 |

*** $p < .001$ (two-sided)

[a] $d = M_1 - M_2 / SD_{pooled}$, calculated using the *cohen.d* function of the R package *psych*

[b] we used Welch’s correction due to unequal variances (as indicated by Levene test for equality of variances, center = median

[c] *p*-value based on 1,000 bootstrapped samples due to severe deviations from the assumption that residuals are normally distributed

Table S4. Planned comparisons (Helmert contrasts): VT-based pedohebephilic men with vs. without convictions for sexual offending (Study 2)

| Variable | Pedohebephilia, no sexual offending (P –SO) | | Pedohebephilia, sexual offending (P +SO) | | P-SO vs. P+SO | | |
|---|---|---|---|---|---|---|---|
| | *M* (*SD*) | *N* | *M* (*SD*) | *n* | *t (df)* | *p* | *d* [a] |
| VT T1 | 2153 (903) | 154 | 2199 (593) | 25 | 0.33 (44.43) [b] | .744 | 0.05 |
| VT T2, 3 | 2557 (791) | 154 | 2459 (825) | 25 | -0.56 (31.58) [b] | .582 | -0.12 |
| VT T4, 5 | 2100 (705) | 154 | 2074 (644) | 25 | -0.19 (34.05) [b] | .854 | -0.04 |
| Age | 33.02 (12.07) | 154 | 42.76 (11.15) | 25 | $3.79^{***}$ (502) | <.001 | 0.82 |
| Height | 178.18 (7.49) | 149 | 177.06 (7.42) | 22 | -0.67 (485) | .506 | -0.15 |
| EHI Laterality Index | 0.73 (0.56) | 154 | 0.86 (0.41) | 25 | 1.12 (502) | .263 | 0.26 |
| ICAR | 8.95 (4.02) | 154 | 7.80 (4.14) | 25 | -1.29 (31.78) [b] | .206 | -0.29 |
| Head Injuries before age 13 | 0.27 (0.55) | 154 | 0.44 (1.00) | 25 | -0.97 (501) | .415 [c] | 0.27 |
| Head Injuries after age 13 | 0.17 (0.52) | 154 | 0.42 (1.02) | 24 | 1.53 (501) | .226 [c] | 0.41 |

$^{***}$ $p < .001$ (two-sided)

[a] $d = M_1 - M_2 / SD_{pooled}$, calculated using the *cohen.d* function of the R package *psych*

[b] we used Welch’s correction due to unequal variances (as indicated by Levene test for equality of variances, center = median)

[c] *p*-value based on 1,000 bootstrapped samples due to severe deviations from the assumption that residuals are normally distributed

*Supplementary Material C: Control analyses based on viewing time.*

**Study 1.** We found no links between age and height for VT-based Pedo+SO ($r$ = -.40, $p$ = .076, 95%*CI* = [-.71, .04]) and Tel-SO ($r$ = -.07, $p$ = .549, 95% *CI* = [-.27, .15]), but height was significantly negatively associated with age for Pedo-SO ($r$ = -.36, $p$ = .014, 95% *CI* = [-.59, -.08]). For head injuries before age 13, we did not find significant links to age for any of the three VT-based groups (Pedo-SO: $r$ = .17, 95%*CI* = [-.01, .41], Pedo+SO: $r$ = .31, 95%*CI* = [-.21, .65], Tel-SO: $r$ = .44, 95%*CI* = [-.11, .24]). We also could not detect links between head injuries after age 13 and age (Pedo-SO: $r$ = -.001, 95%*CI* = [-.25, .32], Pedo+SO: $r$ = .26, 95%*CI* = [-.11, .56], Tel-SO: $r$ = -.06, 95%*CI* = [-.23, .17]). The associated confidence intervals for the head injury measures are based on 1,000 bootstrap samples. Hence, only 1/9 correlations reached significance.

**Study 2.** We detected no links between height (all p < .150), IQ (all $p$ < .475) or head injuries before age 13 (no 95%*CI* based on 1,000 bootstrap samples containing 0) and age for any of the three VT-based groups. For Pedo+SO, we detected a link between age and head injuries after age 13 ($r$ = -.25, 95%*CI* based on 1,000 bootstrap samples = [-.46, -.01], note that the associated confidence intervals are). Hence, only 1/12 correlations reached significance.
